# Supplementary material for: The Role of Dietary Protein in Body Weight Regulation among Active-Duty Military Personnel during Energy Deficit: A Systematic Review
Source: Nutrients. 2023 Sep 12;15(18):3948. doi: 10.3390/nu15183948 (PMC10536394; doi:10.3390/nu15183948)
Supplement: Supplementary file 1 [file nutrients-15-03948-s001.zip › nutrients-2587042-supplementary.pdf]

Table S1. Full Characteristics of included articles (*n* = 8).

Reference, year	Country	Type of Study	Initial Sample (n)	Final sample (n)	Participants	Setting	Time Period	Study length	Age (yrs.)	Protein Dose	EI Measure	EE Measure	Body Weight Measure
Askew et al., 1987 [21]	U.S.A.	Experimental between groups (Pre/Post)	36	34	2nd and 3rd Battalions, Special Forces soldiers during field training exercise	Camp Ethan Allen, VT, U.S.A	Sept 1986 - Oct 1986	30-days	27	112 g/day vs. 64 g/day	Self-Report & nutrient analyses system (USARIEM), calculated from TEEE	Device (ActiGraph), Self-Report (field log)	BW (SECA Scale), FFM & FM (underwater weighing)
Alemany et al., 2008 [24]	U.S.A.	Mixed-Model, Repeated-Measures	36	34	U.S. Marine Corps infantry officer candidates	Military field exercise, Quantico, VA, U.S.A	2006	8-days	24.5 ± 0.3	0.9g/per kg/d vs. 0.5g/per kg/d	Observed (wrappers) & MRE nutrient database, DLW	Device (ActiGraph), & DLW	BW (digital scale), FFM, FM (DXA)
Berryman et al., 2016 [23]	U.S.A.	RCT	68	63	U.S. Marines	(SERE) school, Stone Bay, Camp Lejeune, NC, U.S.A.	Jan 2014- March 2015	45-days	25 ± 3	7g/d, 84g/d, or 133g/d supplement	24-h dietary recall & database analysis, DLW	NM	BW (Digital Scale), FFM, FM (DEXA)
Booth et al., 2003 [1]	Australia	Experimental btw groups	39	37	Airfield defense guards	Exercise Northern Awakening RAAF Base Amberley, U.S.A.	April 1999	12-days	22	63g/d, 88g/d or 116g/d	Observed (wrappers) & database analysis, DLW	Device (ActiGraph)	BW (SECA Scale), FFM & FM (BIA)

Fallowfield et al., 2004 [2]	U.K.	Within-subject, Repeated-measures	249	176	Royal Marines stationed in U.K. for deployment to Afghanistan	6-month deployment Afghanistan	March 2010-October 2010	6-months	28 ± 7	125g/d vs. 95g/d	Self-report (food diary) & analysis, DLW	Device (ActiGraph), Observed-Report, DLW	BW (Scale), Body Comp (skinfolds (eight sites) and circumferential girths (six sites))
Margolis et al., 2014 [26]	Norway	Longitudinal Observational	21	21	Norwegian conscripted soldiers	Minimal military experience (≤2 months)	2012	7-days	20 ± 1	1.59g/kg/d, 1.71g/kg/d	Observed (wrappers & food logs) & analysis, DLW	DLW	BW (Digital Scale)
McAdam et al., 2018 [27]	U.S.A.	Repeated measures, double-blind, parallel groups	69	69	U.S. Army soldiers	One training unit at Fort Benning Georgia, U.S.A.	2017	8-weeks	19 ± 1	0.5g vs. 38.g (2xd protein supplement)	Self-report (diet recall), ED estimation from previous work	NM	BW (Scale), FFM, FM (7-site skinfold)
Pasiakos et al., 2013 [25]	U.S.A.	RCT	42	39	Military personnel from the U.S. Army	Grand Forks, ND, U.S.A.	2012	31-days	21 ± 1	0.8g/kg/d, 1.6g/kg/d or 2.4g/kg/d	Objective (Dietitian/metabolic kitchen prepared meals), indirect calorimetry	Device, indirect calorimetry	BW (digital scale), FFM, FM (DXA)

EI, Energy Intake; EE, Energy Expenditure; BW, body weight; FM, fat mass; FFM, fat-free mass; Body Comp, Body Composition; RCT, randomized controlled trial; SERE, Survival, Evasion, Resistance, Escape; UK, United Kingdom; U.S., United States of America; BIA, bioelectrical impedance analysis; DEXA/DXA, dual x-ray absorptiometry; ED, Energy Deficit; DLW, Doubly Labeled Water, NM, Not Measured

Table S2a. Rating of Study Quality based on Criteria from Downs and Black, 1998.

**Author, Year**	1. Aims	2. Outcomes	3. Participants	4. Interventions	5. Confounders	6. Findings	7. Variability	8. Adverse Events	9. Lost to Follow Up	10. Probability Values	11. Representative - Recruitment	12. Representative - Enrollment	13. Representative - Setting	14. Blinding - Participants	15. Blinding - Assessors	16. Data Dredging	17. Length of Follow Up	18. Statistical Analysis	19. Intervention Compliance	20. Outcome Measures	21. Recruitment - Same	22. Recruitment - Time Period	23. Randomization	24. Randomization Assignment	25. Analysis - Confounding	26. Analysis - Loss to Follow Up	27. Power Analysis
Askew et al., 1987 [21]																											
Alemany et al., 2008 [24]																											
Berryman et al., 2016 [23]																											

Booth et al., 2003 [1]																											
Fallow field et al., 2004 [2]																											
Margo lis et al., 2014 [26]																											
McAd am et al., 2018 [27]														N	U	Y	Y	Y	Y	Y	Y	Y	Y	N			
Pasiak os et al., 2013 [25]																											

**Overall Count**																											
**Yes**	8	8	8	8	5	8	8	5	8	8	1	1	7	3	5	8	8	8	8	8	8	8	5	2	5	8	8
**Unable to Determine**	0	0	0	0	0	0	0	0	0	0	7	7	0	0	1	0	0	0	0	0	0	0	0	2	0	0	0
**No**	0	0	0	0	3	0	0	3	0	0	0	0	1	5	2	0	0	0	0	0	0	0	3	4	3	0	0

**Table S2b.** Description of Study Quality Ratings Provided by Downs and Black, 1998.	
**Supplementary Table Item**	**Downs and Black Rating Prompt**
1. Aims	Is the hypothesis/aim/objective of the study clearly described?
2. Outcomes	Are the main outcomes to be measured clearly described in the Introduction or Methods section?
3. Participants	Are the characteristics of the patients included in the study clearly described?
4. Interventions	Are the interventions of interest clearly described?
5. Confounders	Are the distributions of principal confounders in each group of subjects to be compared clearly described?
6. Findings	Are the main findings of the study clearly described?
7. Variability	Does the study provide estimates of the random variability in the data for the main outcomes?
8. Adverse Events	Have all important adverse events that may be a consequence of the intervention been reported?
9. Lost to Follow Up	Have the characteristics of patients lost to follow-up been described?
10. Probability Values	Have actual probability values been reported (e.g.,0.035 rather than <0.05) for the main outcomes except where the probability value is less than 0.001?
11. Representative – Recruitment	Were the subjects asked to participate in the study representative of the entire population from which they were recruited?
12. Representative – Enrollment	Were those subjects who were prepared to participate representative of the entire population from which they were recruited?
13. Representative – Setting	Were the staff, places, and facilities where the patients were treated, representative of the treatment the majority of patients receive?
14. Blinding – Participants	Was an attempt made to blind study subjects to the intervention they have received?
15. Blinding – Assessors	Was an attempt made to blind those measuring the main outcomes of the intervention?
16. Data Dredging	If any of the results of the study were based on “data dredging”, was this made clear?
17. Length of Follow Up	In trials and cohort studies, do the analyses adjust for different lengths of follow-up of patients, or in case-control studies, is the time period between the intervention and outcome the same for cases and controls?
18. Statistical Analysis	Were the statistical tests used to assess the main outcomes appropriate?
19. Intervention Compliance	Was compliance with the intervention/s reliable?
20. Outcome Measures	Were the main outcome measures used accurate (valid and reliable)?
21. Recruitment – Same Population	Were the patients in different intervention groups (trials and cohort studies) or were the cases and controls (case-control studies) recruited from the same population?

22. Recruitment – Time Period	Were study subjects in different intervention groups (trials and cohort studies) or were the cases and controls (case-control studies) recruited over the same period of time?
23. Randomization	Were study subjects randomized to intervention groups?
24. Randomization - Assignment	Was the randomized intervention assignment concealed from both patients and health care staff until recruitment was complete and irrevocable?
25. Analysis – Confounding	Was there adequate adjustment for confounding in the analyses from which the main findings were drawn?
26. Analysis – Loss to Follow Up	Were losses of patients to follow-up taken into account?
27. Power Analysis	Was a power analysis conducted?
